# Transcranial Magnetic Stimulation Reveals Intrinsic Perceptual and Attentional Rhythms

**DOI:** 10.3389/fnins.2017.00154

**Published:** 2017-03-27

**Authors:** Laura Dugué, Rufin VanRullen

**Affiliations:** ^1^Unité Mixte de Recherche 8242, Centre National de la Recherche ScientifiqueParis, France; ^2^Laboratoire Psychologie de la Perception, Université Paris DescartesParis, France; ^3^Unité Mixte de Recherche 5549, Faculté de Médecine de Purpan, Centre National de la Recherche ScientifiqueToulouse, France; ^4^Centre de Recherche Cerveau et Cognition, Université Paul Sabatier, Université de ToulouseToulouse, France

**Keywords:** attention, non-invasive, oscillations, perception, rhythms, single-pulse, spontaneous, TMS

## Abstract

Oscillatory brain activity has functional relevance for perceptual and cognitive processes, as proven by numerous electrophysiology studies accumulating over the years. However, only within the past two decades have researchers been able to study the causal role of such oscillations using transcranial magnetic stimulation (TMS) technology. Two complementary approaches exist. A majority of research employs rhythmic TMS (rTMS) to entrain oscillatory activity and investigate its effect on targeted brain functions. On the other hand, single pulses of TMS (spTMS) that can be delivered with a high spatio-temporal resolution, can be used to precisely probe the state of the system. In this mini-review, we concentrate on this second approach. We argue that, with no *a priori* hypothesis on the oscillatory frequency of the targeted cortical regions, spTMS can help establish causal links between spontaneous oscillatory activity and perceptual and cognitive functions. Notably, this approach helped to demonstrate that the occipital cortex is periodically involved during specific attentional tasks at the theta (~5 Hz) frequency. We propose that this frequency reflects periodic inter-areal communication for attentional exploration and selection. In the future, clever combination of non-invasive recording and stimulation with well-controlled psychophysics protocols will allow us to further our understanding of the role of brain oscillations for human brain functions.

## Introduction

Since the early ages of neuroimaging, researchers have devoted their energy to understanding the relation between brain activity and sensory experience. Using invasive recording in animal models, such as monkeys or mice, they learned a great deal about the behavior of single neurons, populations of neurons (Buzsáki et al., 2012), and the link between their activity and behavioral processes. Technological advances allowed neuroscience research to move toward a better understanding of human brain functions by employing non-invasive, *in vivo* recordings. Recording neural brain activity using electro/magneto-encephalography (EEG/MEG) is particularly productive, allowing one to characterize, with high temporal resolution, the dynamics of the neural substrates of specific perceptual and cognitive functions.

Numerous researchers support the idea that neural oscillations dynamically modulate perception and cognition (Thut et al., 2012; VanRullen, 2016). This topic gathers increasing interest in the scientific community while compelling evidence accumulates. A variety of brain functions, such as attention (e.g., Busch and VanRullen, 2010; Dugué et al., 2015a, 2016; Landau et al., 2015), perception (e.g., Ergenoglu et al., 2004; Busch et al., 2009; Romei et al., 2010; Dugué et al., 2011b), and memory (e.g., Jensen et al., 2002; Bonnefond and Jensen, 2012), have been correlated to oscillations in specific brain areas and frequency bands. The question remains, however, as to whether these oscillations are a mere by-product of brain processing, or carry functional meaning and constitute the neural substrate underlying perception and cognition.

To address this question, one can focus on spontaneous oscillatory activity and its predictive nature for subsequent processing (VanRullen et al., 2011; VanRullen, 2016, in press). If neural oscillations have a specific, functional role, then it is possible to link their spectral properties (frequency, phase, and amplitude) to perceptual and/or cognitive processes assessed via behavioral measurements. However, were such a link established, it would be correlational. Non-invasive brain stimulation (NIBS) has recently provided a way to move beyond correlational analyses and establish a causal relation between neuronal excitability and brain processes (Silvanto and Muggleton, 2008; Thut and Miniussi, 2009; Thut and Pascual-Leone, 2010; Thut, 2014) with good spatial and temporal resolution. This mini-review considers experiments in which single-pulse Transcranial Magnetic Stimulation (spTMS), a NIBS methodology with high spatial and temporal resolution, is used to probe spontaneous oscillations, and establish a causal link with perceptual and cognitive processes (Miniussi et al., 2013 for other NIBS approach). We compare this approach to the rhythmic-TMS (rTMS) approach in which brain activity is entrained at a predefined oscillatory frequency to evaluate its causal role on subsequent performance, and argue that it can bring additional information regarding the role of spontaneous, oscillatory activity on perceptual and cognitive processes.

## Rhythmic non-invasive brain stimulation

In 1985, Barker and colleagues first demonstrated that non-invasive magnetic stimulation of the motor cortex can produce a Motor Evoked Potential (MEP), thus probing neuronal excitability of the targeted area (Barker et al., 1985). This was the starting point of an extensive literature on the use of magnetic pulses to study motor physiology, and, later, cognition and perception (Wassermann et al., 2008). Amassian first proposed that spTMS could act as a reversible, virtual lesion (Amassian et al., 1989), and thus inform the role of given regions in particular functions. Later on, rTMS was applied off-line (i.e., before assessment of TMS-induced neural changes) to specific brain regions in order to increase (with high frequency stimulation) or decrease (with low frequency stimulation; <1 Hz) spontaneous excitability, and thus alter specific functions (Pascual-Leone et al., 1998; Kobayashi and Pascual-Leone, 2003). rTMS also became a candidate treatment of brain disorders associated with abnormal synchronization of neural ensembles such as autism, Parkinson, or schizophrenia (Uhlhaas and Singer, 2006). Positive treatment outcomes suggest that synchronizing (or desynchronizing) neural activity could help to treat some of these pathologies. For example, entraining oscillatory activity in the motor cortex would modulate motor functions (Joundi et al., 2012). rTMS has thus become a promising alternative to pharmacological or electroconvulsive therapy, especially in cases of medication-resistant disorders.

More recently, rTMS was used online, i.e., concurrently with perceptual tasks, to interact with, or induce oscillatory activity in the stimulated region, for investigating the role of such oscillation, at particular frequencies, on subsequent performance. Chanes et al. recently showed that a 4-pulse pattern, at specific frequencies (30 and 50 Hz), applied over the right frontal eye field (FEF), enhances performance in a visual detection task. Interestingly, when a 4-pulse pattern, covering the same time-window, but with no frequency specificity (random pattern), is applied over the same region, no such effect is observed (Chanes et al., 2013). The authors suggest that rhythmic stimulation patterns are able to synchronize neural activity at particular frequencies, and infer a link between neural oscillations and perceptual performance. However, because brain activity was not recorded during the stimulation, the conclusion is speculative. The first study to successfully demonstrate the ability of rTMS to entrain oscillatory activity in visual areas is by Thut et al. (2011). They applied rTMS over the parietal cortex at the individual alpha peak frequency (~10 Hz) while simultaneously recording brain activity using a TMS-compatible EEG (Thut et al., 2011). They observed that the stimulation is able to produce, and progressively enhance, parietal alpha activity, and that this effect is dependent on the phase of ongoing alpha oscillations prior to the stimulation. Similarly, studies have shown that rTMS can entrain beta oscillations in non-visual areas such as prefrontal cortex (Hanslmayr et al., 2014) and motor cortex (Romei et al., 2015). These results confirm that rTMS is able to entrain ongoing neural oscillations, consequently impacting brain functions and subsequent perceptual performance (Romei et al., 2016 for a comprehensive review).

This mini-review focuses on visual and motor systems. However, rTMS has been applied to other systems, e.g., auditory and somatosensory systems, but to a lesser extent. This can be due to multiple factors such as: (1) the visual and motor systems have the advantage, when stimulated, to provide easily observable outcomes (phosphene and motor response, respectively) that directly reflect cortical excitability; (2) visual cortex and motor cortex are large regions that can be easily mapped; (3) stimulation of the auditory cortex is uncomfortable for the participants (close to the ear and nerves innervating the face); (4) the rTMS itself induces somatosensory sensations making it difficult to draw conclusions when stimulating the sensorimotor area.

The rTMS approach has proven useful to link oscillations in given brain areas, and at particular frequencies, and cognitive or perceptual processes. Yet, it occludes information about the ongoing state of the system and the role of spontaneous oscillations on brain functions. Additionally, to perform such an experiment, *a priori* hypotheses about the stimulation frequency to apply to the cortical region are necessary. In the next section, we argue that TMS in its single-pulse form (spTMS) can be used to probe the intrinsic state of the system and its role on brain functions.

## TMS to probe intrinsic rhythmicity of perception and attention

In the past decade, accumulating evidence has demonstrated the predictive nature of the spectral components of ongoing oscillations (i.e., phase and amplitude) on perceptual processes (VanRullen et al., 2011; VanRullen, 2016, in press). Interestingly, TMS, and in particular spTMS due to its high temporal and spatial resolution compared with other stimulation techniques such as tACS/tDCS (Paus et al., 2001; Miniussi et al., 2013), can be used to characterize spontaneous activity and its role on perceptual processes. Here, we review three ways in which spTMS has been used to do so.

First, spTMS helped characterize the natural frequency of spontaneous oscillations. Applied over three distinct cortical areas while recording EEG activity and in the absence of perceptual task, spTMS consistently evokes alpha oscillations (8–12 Hz) in occipital cortex, low beta (13–20 Hz) in parietal cortex and high beta (21–50Hz) in frontal cortex, even when these areas are not stimulated directly by sensory information (Rosanova et al., 2009). This result suggests that TMS can be used to probe spontaneous oscillations, i.e., the pulse triggers the system to resonate at its natural, intrinsic frequency.

Secondly, spTMS has been used to probe spontaneous neuronal excitability. Specifically, several research groups, including ours, have observed that pre-TMS, alpha oscillations affect the outcome of the stimulation. In a TMS-EEG study, spTMS was applied over the primary motor cortex to induce a motor evoked potential (MEP; Sauseng et al., 2009; van Elswijk et al., 2010). Researchers observed that MEP generation is facilitated by low alpha amplitude prior to the stimulation. spTMS has also been administered to the parieto-occipital pole to induce phosphene perception (illusory flash retinotopically located). Studies have shown that phosphene perception is dependent on both the amplitude (Romei et al., 2008) and phase (Dugué et al., 2011b; Romei et al., 2012) of pre-stimulation alpha oscillations. In a nutshell, both alpha amplitude and phase are good indicators of cortical excitability. Together, these results suggest that ongoing alpha oscillations play a causal role in cerebral processes, and can be successfully probed using spTMS.

**Figure 1:**
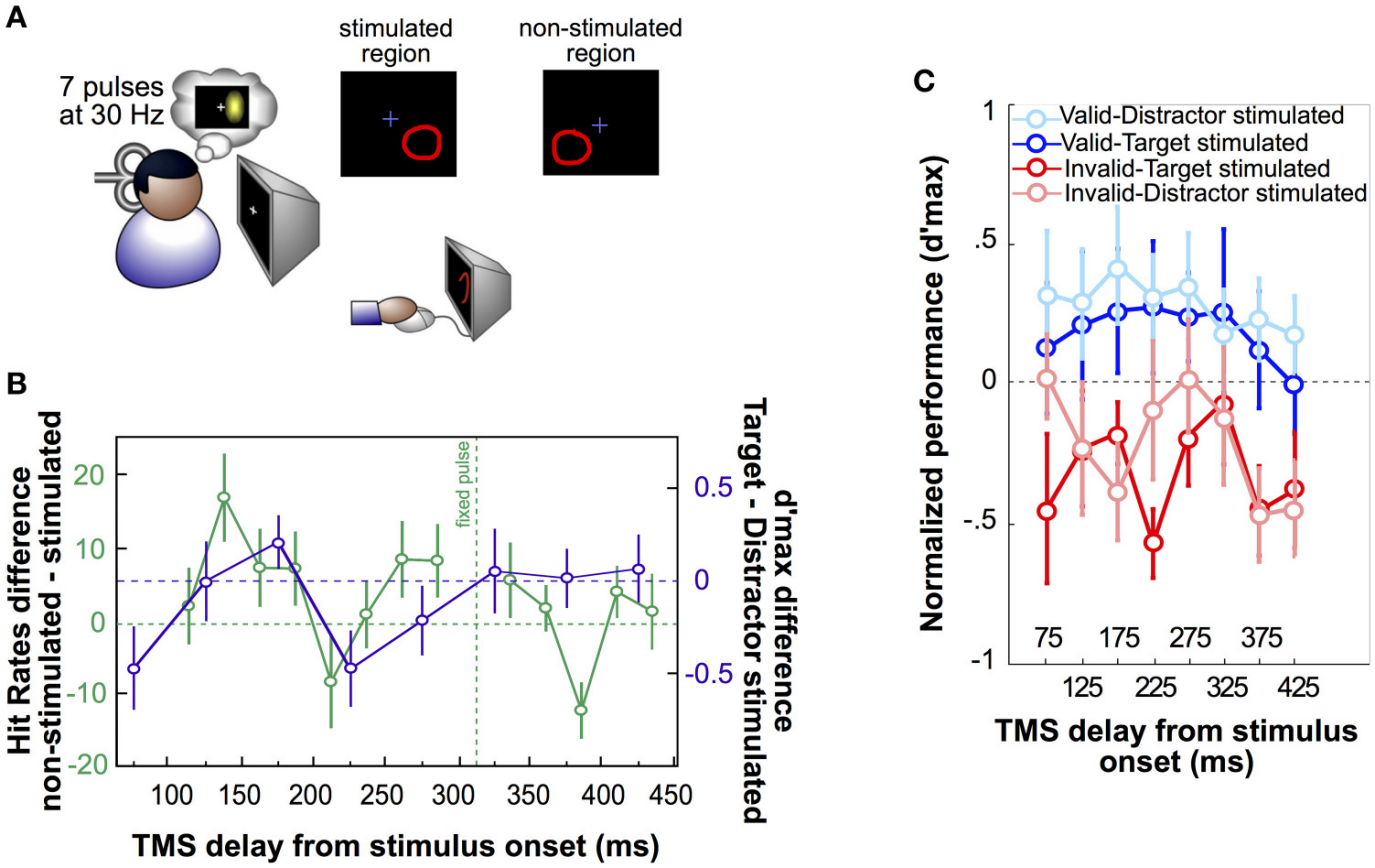
**Single-pulse TMS applied to specific retinotopic regions. (A)** Phosphene mapping procedure, first developed by Dugué et al. (2011a). Seven pulses of TMS are applied over the occipital pole (left or right V1/V2) to induce the perception of a phosphene (illusory flash; either on the right or the left visual field). Observers are tasked to draw the phosphene on the screen using the computer mouse. The phosphene region, so called “stimulated region,” is then used to present visual stimuli, whereas the symmetric, “non-stimulated” region is used as specific, retinotopic control. This approach has been applied to obtain the results represented in **(B,C)**. **(B)** In Dugué et al. (2015a), observers performed a difficult search task in which they had to look for a T among L distractor letters. Stimuli (always four in the search array) were either presented in the stimulated or the non-stimulated region (green curve). A pulse of TMS was consistently applied ~300 ms, a delay known to interfere with this specific search (Dugué et al., 2011a), after the onset of the search array (fixed pulse) while another pulse was applied at various delays around the first one. Positive values in the hit rate difference between non-stimulated and stimulated trials represent an impairment of search performance by TMS, while negative values correspond to performance facilitation. As confirmed by complementary analyses, the hit rate fluctuations as a function of TMS delay is periodic at 5.7 Hz (theta). The purple curve corresponds to the difference of the pink and red curves from panel **C**, and is also periodic at 5 Hz (theta). **(C)** In Dugué et al. (2016), observers performed a 2-AFC orientation discrimination task in which voluntary attention was manipulated using valid (in blue) or invalid (in red) cueing. Performance was measured as per d'max (d' at asymptotic performance). Two Gabor patch stimuli were presented simultaneously on the screen. Dark color plots represent trials in which the target (Gabor patch for which the observers had to report the orientation) was in the stimulated region, while light color plots correspond to trials in which the distractor was stimulated. In each trial, a double pulse of TMS (25 ms interval) was applied at various delays after the stimuli display onset. As confirmed by complementary analyses, performance in the invalid condition (during which the observers have to reorient their attention to the opposite stimulus location) fluctuates periodically at 5 Hz (theta; see purple curve in panel **B**). Additionally, this modulation is shifted in phase between the invalid-target stimulated and invalid-distractor stimulated conditions.

Thirdly, spTMS applied at multiple delays during a behavioral task was used to characterize the temporal dynamics of stimulated cortical areas in specific cognitive processes. Using a phosphene mapping procedure (Dugué et al., 2011a), TMS was administered over the occipital pole to interact with the retinotopic area relative to the visual stimulus presentation (Figure 1A) while observers performed an attentional search task (Dugué et al., 2015a). The results displays a periodic pattern of interference at ~6 Hz (theta) in the behavioral performance (Figure 1B), independently associated with a periodicity in the EEG signal at the same frequency. Applying TMS at various intervals after search onset, this study demonstrates that attentional search is modulated periodically by brain oscillations. More recently, following a similar procedure (Figure 1A; Dugué et al., 2016), TMS was used to probe theta phase-reset induced by attentional reorienting to task relevant stimuli (Figure 1C). By stimulating at various delays while observers performed a 2-AFC orientation discrimination task, this study demonstrates that reorientation of voluntary attention periodically involves occipital areas at 5 Hz (theta).

Applying spTMS at various intervals in order to investigate the temporal dynamics of given processes comes at the cost of temporal resolution. Indeed, such studies require the sampling of multiple delays during the perceptual task, but given experiment constraints, it is challenging to sample more than a dozen of time points. Consequently, these studies can only characterize low frequency bands, typically below ~20 Hz. It can be argued that a brief visual stimulus can be similarly used to characterize the periodicity of cognitive processes, but without the concern of temporal resolution. Yet, spTMS provides a powerful, non-invasive tool allowing direct readout of neuronal excitability, in a focal cortical region and at precise points in time (e.g., Romei et al., 2010; Dugué et al., 2011b).

Contrary to the use of rTMS to entrain neuronal oscillations to investigate their causal role on cognitive processes, spTMS can be used as a probing mechanism of intrinsic rhythmic processes. This method allows researchers to reveal periodicity with no *a priori* hypothesis on the specific frequency of the targeted system. The last section describes an observation we made while reviewing the spTMS literature, and speculations about the underlying process.

## A tentative model of the role of alpha and theta oscillations in sensory sampling and inter-areal communication

So far we have argued that spTMS can be used as a complementary approach to rTMS to temporally probe the state of the brain. We also showed that when stimulating over retinotopic visual areas, one can specifically interact with a given part of the visual environment and compare the behavioral effects with specific, retinotopic controls (Figure 1A). We describe here how such an approach revealed a possible link between inter-areal communication and rhythmic attentional exploration, and propose a tentative model of the role of alpha and theta oscillations in such process.

Recently, in a comprehensive review of the oscillation literature, VanRullen reported that two types of perceptual rhythms can coexist in the visual domain: an alpha rhythm (~10 Hz) that would be associated with sensory processes, and a theta rhythm (~7 Hz) that would be associated with attentional sampling (VanRullen, 2016, in press). spTMS studies reported in the previous section offer to extend this observation. Used as a probe of spontaneous neuronal excitability in the early visual cortex, spTMS reveals that occipital alpha oscillations (i.e., the natural frequency of this area) can predict the outcome of given perceptual processes. Critically, when attentional exploration is involved, the rhythmicity observed is, instead, at the theta frequency. In Dugué et al. (2015a) observers had to monitor the visual environment in a difficult search task in order to find a target among similar looking distractors. In Dugué et al. (2016), voluntary attention was manipulated using a central visual cue to either a valid (same location as the target) or invalid (opposite location) stimulus location. In invalid trials, observers had to spatially reorient their attention to the opposite target location. In both experiments, the stimulation of the occipital cortex (V1/V2) induced periodic fluctuations of performance, even at late delays after the onset of the visual stimuli. This suggests that feedback connections between high-level control regions (e.g., FEF) and the occipital cortex were involved to allow attentional exploration and selection. This inter-areal communication has been hypothesized through hierarchical frameworks in which low-level areas, presumably the early visual cortex, decompose the visual input into distinct features, while high-level region(s) perform attentional selection. Interactions between the former and the latter would then control attentional exploration (e.g., Treisman, 1998; Itti and Koch, 2001; Deco et al., 2002; Juan and Walsh, 2003; Saalmann et al., 2007; Dugué et al., 2011a, 2015a, 2016).

Interestingly, theta periodicity has been observed in various studies in which attentional exploration is taking place, potentially involving such feedback connections (VanRullen et al., 2007; Busch and VanRullen, 2010; Landau and Fries, 2012; Fiebelkorn et al., 2013; VanRullen, 2013; Song et al., 2014; Dugué et al., 2015b; Huang et al., 2015; Landau et al., 2015; Dugué et al., in press). More recently, van Diepen et al. (2016) used a three-alternative forced choice task to manipulate spatial attention. They observed alpha-lateralization (higher alpha amplitude in the ipsilateral region to the cued location) when the target was paired with a low-similarity distractor, but increased theta amplitude when target and distractor were highly similar. These results motivate us to speculate further that alpha and theta oscillations can coexist after display onset, but that theta oscillations dominate when attentional exploration is needed to perform the task at hand.

Yet, it is only through the stimulation of the occipital cortex that a link between the oscillatory modulation of performance and inter-areal communication can be proposed. TMS allows revealing specific moments at which the targeted cortical area is critical for information processing. Here, these moments correspond to the specific timings at which the occipital cortex receives feedback from higher-order, attentional areas. Alternatively, it can readily be argued that the effect described above could be observed with a feedforward-only model, i.e., late effects of spTMS over the occipital cortex do not prove the presence of feedback. It is possible that the time-points of TMS vulnerability correspond to time-points of higher-order readout of information coming from early visual regions, e.g., through phase coupling between (occipital) alpha and (higher-order) theta oscillations. However, there is good evidence that attention acts via feedback to sensory areas. First, these feedback connections have been described in various models of attention (e.g., Tsotsos et al., 1995; Spratling and Johnson, 2004; Hamker and Zirnsak, 2006; Reynolds and Heeger, 2009; Miconi and VanRullen, 2016), and proposed to modulate the processing of sensory information by early visual areas (e.g., Chelazzi et al., 1993; Motter, 1994; Luck et al., 1997; Mehta et al., 2000; Schroeder et al., 2001; Treue, 2001; Kastner and Pinsk, 2004; Saalmann et al., 2007; Bressler et al., 2008). Second, if there is indeed feedback during attentional exploration, and if these feedback are periodic in the theta frequency range, they would then periodically reset the signal in the receiving areas, i.e. alpha oscillations in the early visual regions, and thus induce theta oscillations in these regions as well. Finally, in Dugué et al. (2015a), occipital EEG activity is measured during a visual search task and an increase in the power and the phase-locking of theta oscillations, and not alpha oscillations, is observed. Considered together, these aforementioned studies motivate our choice to speculate about feedback attentional signals, and propose this tentative model of inter-areal communication and attentional exploration.

**Figure 2:**
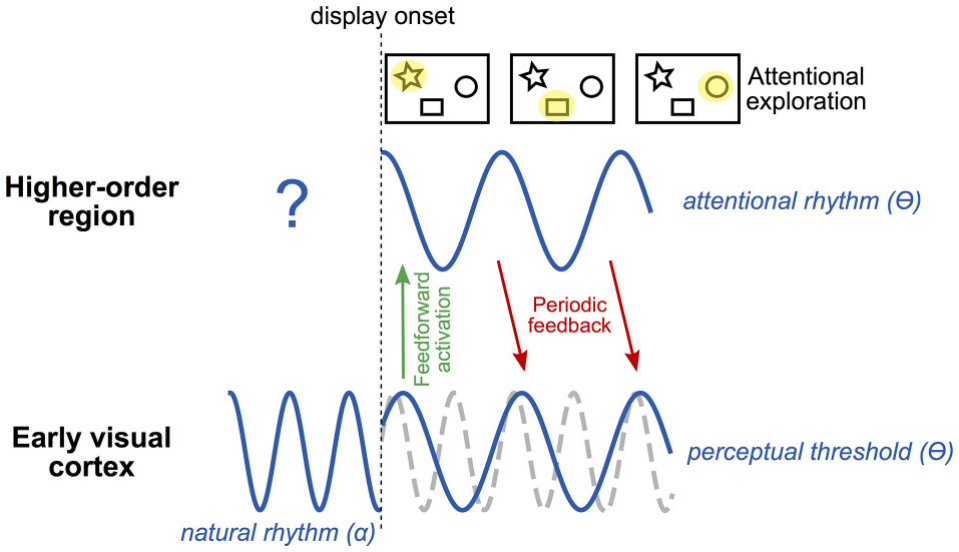
**Tentative model of rhythmic attentional exploration**. The early visual cortex naturally oscillates at the alpha frequency (~10 Hz). At the onset of a visual display involving attentional exploration, early visual cortex sends a first, feed-forward wave of activation to higher-order regions. Among these regions, at least one is involved in producing an attentional rhythm. This region will send periodic feedback to early visual cortex along with attentional exploration; i.e., at each cycle of the attentional rhythm, the selection of a (potentially) different stimulus (or group of stimuli) will allow the exploration of the visual scene by attention. The attentional rhythm, most likely at the theta frequency (~5 Hz), will impose a periodicity to the perceptual threshold at the same frequency. The natural alpha frequency (whose phase may be partially reset by stimulus onset) would thus coexist with the theta attentional rhythm, and either be recordable concurrently or be masked depending on task relevance. Note that, depending on task demands and the participant's strategy, the attentional rhythm in higher-order regions may already be active before display onset (in an anticipatory manner) and potentially influence early visual regions by periodic feedback.

In summary, spTMS applied at various delays over the occipital pole can reveal rhythmicity in the communication between low and high-level areas necessary for attentional exploration and selection. We hypothesize that such periodicity, most likely at the theta frequency, is measurable when inter-areal communication is needed, i.e., during an attentional exploration task, and comes in addition to the natural frequency (occipital alpha; Figure 2). The corollary to this proposal is that theta rhythmicity would also be revealed in high-level, control regions involved in sending feedback to occipital areas permitting attentional selection. This hypothesis could be tested by applying spTMS at multiple time intervals over a high-level control region (e.g., FEF) and measuring behavioral consequences during a task involving attentional exploration and selection.

## Summary and future directions

TMS is increasingly used in the scientific community since it offers a unique way to establish causal relations between brain functions and cerebral activity non-invasively in humans. rTMS can be used to entrain brain activity at specific frequencies to investigate the role of the entrained oscillation for given perceptual and cognitive processes. spTMS is based on the opposite principle. Instead of entraining targeted oscillations, it can agnostically probe the intrinsic state of the brain. This is useful when one has no *a priori* hypothesis regarding the frequency of the oscillation of interest. spTMS is, however, uncommon in the oscillation literature given the necessary trade-off between temporal resolution and the amount of trials necessary for statistical analyses. In the future, one will need to find the sweet spot for well-controlled psychophysics designs and appropriate non-invasive stimulation and recording techniques.

## Author contributions

LD and RV conceived the review focus and reviewed the literature. LD summarized the literature review and wrote the first draft. LD and RV finalized the manuscript and approved the final version of the manuscript.

## Funding

This work was supported by an ERC Consolidator grant P-CYCLES number 614244 to RV.

### Conflict of interest statement

The authors declare that the research was conducted in the absence of any commercial or financial relationships that could be construed as a potential conflict of interest.
